# Survival of *Escherichia coli* O157:H7 in Soils from Jiangsu Province, China

**DOI:** 10.1371/journal.pone.0081178

**Published:** 2013-12-02

**Authors:** Taoxiang Zhang, Haizhen Wang, Laosheng Wu, Jun Lou, Jianjun Wu, Philip C. Brookes, Jianming Xu

**Affiliations:** College of Environmental and Natural Resource Sciences, Zhejiang Provincial Key Laboratory of Subtropical Soil and Plant Nutrition, Zhejiang University, Hangzhou, China; Beijing Institute of Microbiology and Epidemiology, China

## Abstract

*Escherichia coli* O157:H7 (*E. coli* O157:H7) is recognized as a hazardous microorganism in the environment and for public health. The *E. coli* O157:H7 survival dynamics were investigated in 12 representative soils from Jiangsu Province, where the largest *E*. *coli* O157:H7 infection in China occurred. It was observed that *E. coli* O157:H7 declined rapidly in acidic soils (pH, 4.57 – 5.14) but slowly in neutral soils (pH, 6.51 – 7.39). The survival dynamics were well described by the Weibull model, with the calculated *t_d_* value (survival time of the culturable *E. coli* O157:H7 needed to reach the detection limit of 100 CFU g^−1^) from 4.57 days in an acidic soil (pH, 4.57) to 34.34 days in a neutral soil (pH, 6.77). Stepwise multiple regression analysis indicated that soil pH and soil organic carbon favored *E. coli* O157:H7 survival, while a high initial ratio of Gram-negative bacteria phospholipid fatty acids (PLFAs) to Gram-positive bacteria PLFAs, and high content of exchangeable potassium inhibited *E. coli* O157:H7 survival. Principal component analysis clearly showed that the survival profiles in soils with high pH were different from those with low pH.

## Introduction

Applications of animal manure as fertilizers or soil amendments to agricultural soils are routine, world-wide. In the UK, the annual amount of animal manure applied to land was recently estimated at 4.3×10^5^ tons dry weight [1]. Though animal manure can provide nutrients, a variety of pathogenic bacteria may survive in the manure, which in turn may serve as a primary hazardous material for environmental contamination and as a public health threat [2]. For example, *Escherichia coli* O157:H7 (*E*. *coli* O157:H7), which can cause severe hemorrhagic colitis and haemolytic uraemia in humans, can persist in soil for days to more than 1 year following manure application to land [3]. It was reported that 20 people were infected with *E*. *coli* O157:H7 through manure-contaminated soil after camping on a field in Scotland that was previously grazed by sheep [4]. Increasing evidence shows that soil and animal manures are the main transport agents of *E*. *coli* O157:H7 to contaminate fresh vegetables, fruits and drinking water [1], [5], [6]. Therefore, it is important to understand the nature of *E*. *coli* O157:H7 survival and its infective risk in soil or soil-related (manure) environments.

In America, about 63,000 human cases of *E*. *coli* O157:H7 infections have been reported every year [7]. Many studies have focused on the survival of *E*. *coli* O157:H7 in soil, manure, water, and vegetables [1], [2], [4], [5], [8], [9]. However, very little attention has been paid to *E*. *coli* O157:H7 survival and its potential environmental contamination risk in the soils in the areas where outbreaks of *E. coli* O157:H7 infection have occurred. Previous studies found that the survival time of *E*. *coli* O157:H7 in soils depends on soil type, physicochemical properties, indigenous microorganisms, etc. [2], [9]–[15]. Franz et al. (2008) pointed out that higher amounts of dissolved organic carbon and dissolved organic nitrogen were the best predictors for long *E*. *coli* O157:H7 survival time in organically managed soils [2]. In several experiments, *E*. *coli* O157:H7 persisted longer in silty clay soils than in sandy soils [2], [10], [11]. *E*. *coli* O157:H7 survived for up to 77, 226, and 231 days at 5, 15, and 25°C in manure-amended autoclaved soil, respectively [13]. *E*. *coli* O157:H7 survived significantly longer under anaerobic than under aerobic conditions in manure and slurry [14]. Yao et al. (2012) and van Elsas et al. (2012) proved *E*. *coli* O157:H7 survival was affected by indigenous microorganisms in soil [9], [15]. Different *E*. *coli* O157:H7 survival rates indicate the different potential risks of the pathogen contamination under various soil environments. Consequently, a better understanding of *E*. *coli* O157:H7 survival in soils will help in reducing the potential risk of pathogen contamination and avoiding human infection from the pathogen.

In the present study, experiments were carried out to investigate *E*. *coli* O157:H7 survival in 12 soils taken from Jiangsu Province, China. In 1987, researchers firstly detected *E*. *coli* O157:H7 from the fecal samples of patients in Jiangsu Province, where the largest *E*. *coli* O157:H7 outbreak in China occurred [16], [17]. Later, Xu et al.(1990) found that the biochemical reactions of five strains of *E. coli* O157:H7, which were isolated from 486 stool specimens of patients with diarrhea in Xuzhou City, Jiangsu Province, were almost identical with those of the well-known *E. colt* O157:H7 (strain EDL933) [18]. Recently, numerous researchers reported that *E*. *coli* O157:H7 (strain EDL933) had been detected in excrement, sewage, foods, and soils from many provinces of China, including Jiangsu Province [16], [19]–[21]. Thus, *E*. *co*li O157:H7 (strain EDL933) was selected as a representative strain in this study.

Most *E*. *coli* O157:H7 outbreaks occur in summer [17]–[19]. The summer mean temperature in Jiangsu Province is about 26°C [22]. Furthermore, the water content under –33 kPa, indicating the water holding capacity of the soil and representing the highest available water contents in soil, was generally used to simulate field conditions [23]. Hence, our simulation experiments also used incubation conditions of 25±1°C and water content under –33 kPa. The aims of this study were to (1) investigate *E*. *coli* O157:H7 survival dynamics in soils, (2) identify the relationships between *E*. *coli* O157:H7 survival time and soil physicochemical properties and microbial community structure, and (3) understand the possible risks of pathogen contamination to prevent further disease outbreaks from *E*. *coli* O157:H7.

## Materials and Methods

### Ethics Statement

The samples were not collected from national parks, protected areas or private land. Hence, no specific permission was required to obtain these samples. The sampling did not cause any disturbance to the environment or to protected species at the sampling sites.

### Soils

The 12 soils (S1–S12) used in this study were taken from Jiangsu Province, China (32.05^o^N – 34.70^o^N). Three replicates were sampled at each soil site from the surface horizon (0–20 cm) and each sample was a composite of several individual soil cores taken at 5-m interval. After sampling, the 36 individually bulked fresh soil samples were immediately taken to the laboratory in coolers containing ice packs. The samples were then hand-picked to remove discrete plant residues, sieved <2 mm, homogenized thoroughly, and then stored at 4 °C. According to the protocols of the Agricultural Chemistry Committee of China [24], a sub-sample of the sieved soil from each sample was air-dried for physical and chemical analyses, including pH, total organic carbon (OC), exchangeable potassium (K), humic acid, fulvic acid, sand, clay and silt. Total nitrogen (TN) was determined following digestion using a Kieldahl apparatus (BÜCHI Labortechnik AG, Flawil, Switzerland). Total dissolved organic carbon (DOC) and total dissolved nitrogen (TDN) were measured by a Multi N/C TOC analyzer (Analytic Jena AG, Jena, Germany). Exchangeable potassium (K) was extracted with 1 M ammonium acetate and measured by Flame Atomic Absorption Spectrophotometry (Analytik Jena AG, Jena, Germany). The soil water content at –33 kPa was determined using a pressure membrane apparatus (Soil Moisture Equipment Corp, CA,USA) as described by Richards [23].

Another portion of each sieved fresh sample was frozen at –80°C and then freeze-dried (FreeZone Freeze Dry Systems LABCONCO Corp, MO, USA) for phospholipid fatty acids (PLFAs) analysis [25]. The PLFAs are the specific components of cell membranes that are only found in intact (viable) cells [26]. Many studies have widely used PLFAs to express the biomass and composition of microbial communities in soils [9], [27], [28]. Thus, PLFAs were used to determine the effects of soil indigenous microorganisms on *E*. *coli* O157:H7 survival in this study. According to Ding et al. (2009) [29] and Ying et al. (2013) [30], the PLFA biomarkers i15:0, a15:0, 15:0, i16:0, 16:1ω7c, i17:0, a17:0, cy17:0, 17:0, 18:1ω7c, cy19:0ω8c were used to represent bacterial biomass; i15:0, a15:0, i16:0, i17:0, and a17:0 were used to indicate Gram-positive (G^+^) bacteria biomass; 16:1ω7c, 18:1ω5c, cy17:0, and cy19:0 indicated Gram-negative (G^−^) bacterial biomass. Polyenoic, unsaturated PLFA 18:2ω6,9c was used to represent fungal biomass. The fatty acids 10Me 16:0, 10Me 17:0, 10Me 18:0 were used to indicate actinomycetic biomass. Based on the studies of the Institute of Soil Science, Academia Sinica [31], the 12 soils used were classified into acidic soils (pH < 6.5) and neutral soils (pH > 6.5). Selected soil properties are shown in Table 1.

**Table 1:** Physical, chemical, and biological properties of the soils used in this study.

Soil Code	Location	pH	OC#	TN	C/N	K	DOC	TDN	FA	HA	HA/FA	PLFA-T	PLFA-F	PLFA-A	PLFA-B	PLFA-G^-^	PLFA-G^+^	G^−^/G^+^	Clay	Silt	Sand
			g kg^−1^			mg.kg^−1^			%			n mol g^1^							%		
S1	Lianyungang	4.57	20.3	1.51	13.44	89.10	51.97	7.46	6.40	43.25	6.75	35.83	0.58	4.24	16.63	7.29	7.67	0.95	17.13	16.72	66.15
S2	Xuzhou	7.27	25.4	2.44	10.41	194.86	28.55	14.97	6.26	22.28	3.56	67.19	1.68	8.52	22.95	10.47	10.77	0.97	31.73	35.19	33.08
S3	Xuyi	6.77	34.4	3.14	10.96	207.22	88.06	6.70	4.68	17.41	3.72	125.46	1.61	14.61	42.25	13.12	27.47	0.47	40.52	32.74	26.74
S4	Yangzhou	4.90	11.6	1.24	9.35	53.72	29.41	2.94	7.67	51.72	6.74	38.45	1.09	4.31	15.71	7.70	7.75	0.99	23.51	48.91	27.58
S5	Nanjing	6.61	8.1	1.01	8.02	123.16	19.83	5.74	23.09	16.91	0.73	21.39	0.21	2.75	9.63	2.50	5.69	0.43	41.12	39.69	19.19
S6	Nanjing	7.39	15.2	1.63	9.32	106.51	30.64	10.05	21.05	3.36	0.16	85.11	1.85	10.31	37.54	19.96	16.71	1.19	35.69	34.66	29.65
S7	Yangzhou	5.14	16.1	1.71	9.42	144.06	61.28	5.66	8.88	38.57	4.34	43.93	1.17	5.43	19.68	8.83	10.62	0.83	27.48	44.04	28.48
S8	Jurong	5.03	15.4	1.42	10.85	98.37	42.58	3.41	11.88	37.14	3.12	46.21	1.31	5.37	18.75	9.50	8.94	1.06	33.77	40.24	25.99
S9	Nanjing	4.66	51.5	3.40	15.15	165.56	134.27	29.42	18.46	16.42	0.89	106.54	1.64	10.91	44.16	22.62	20.81	1.09	18.01	39.13	42.86
S10	Nanjing	6.51	13.7	1.30	10.54	67.67	26.85	3.70	5.62	14.38	2.56	27.91	0.42	3.45	11.87	4.71	6.14	0.76	20.57	47.80	31.63
S11	Jiangyin	4.68	19.4	1.43	13.56	108.82	33.67	5.49	7.16	43.40	6.05	26.6	0.91	3.47	11.67	5.50	6.01	0.92	33.06	39.91	27.03
S12	Jurong	6.78	61.4	5.00	12.28	325.53	115.06	14.27	4.30	20.99	4.89	121.5	3.40	23.21	44.41	16.48	25.9	0.63	24.61	40.51	34.88

# Total soil organic carbon (OC); total soil nitrogen (TN); the ratio of OC to TN (C/N); exchangeable potassium (K); dissolved organic carbon (DOC); total dissolved nitrogen (TDN); the ratio of fulvic acid to organic carbon (FA); the ratio of humic acid to organic carbon (HA); total phospholipid fatty acids (PLFA-T); fungus phospholipid fatty acids (PLFA-F); actinomyces phospholipid fatty acids (PLFA-A); bacteria phospholipid fatty acids (PLFA-B); Gram-negative bacteria phospholipid fatty acids (PLFA-G^-^); Gram-positive bacteria phospholipid fatty acids (PLFA-G^−^); the ratio of PLFA-G^−^ to PLFA−G^+^ (G^−^/G^+^);

### Incubation experiments

The preparation of *E*. coli O157:H7 (strain EDL933) and inoculums were described previously by Wang et al. (2013) [32]. *E. coli* O157:H7 cells in sterile deionized water were inoculated into soils to achieve a cell density of about 10^7^ colony-forming units per gram soil oven-dry weight (CFU g^−1^). The incubation experiments were conducted on each of the three soil replicates taken from each of the twelve sampling sites (a total of 36 soil samples). All the inoculated and uninoculated control soil samples were incubated in the dark at 25±1°C and kept at soil moisture of –33 kPa.

The inoculated soils were sampled at 0, 0.25, 1, 2, 3, 5, 7, 10, 15, 20, 25, 30 and 35 days after inoculation (DAI), and *E*. *coli* O157:H7 was extracted by 0.1% peptone buffer (Lab M, Lancashire, UK). The resulting soil suspension was then subjected to 10-fold serial dilutions and *E*. *coli* O157:H7 enumerated at the last three of the serial dilutions. The detection limit of the plating technique is about 100 CFU g^−1^. The sampling was stopped after plate counts of zero appeared twice in succession during the incubation.

### Statistical analysis

The survival data was converted to log_10_ (CFU g^−1^) and then analyzed by the Weibull survival model (Equation 1), as described by Wang et al. (2013) [32] and Ma et al. (2013) [33].



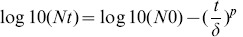
(1)


*N_t_* represents the number of surviving cells remaining at time *t*, *N*_0_ is the initial cells of the inoculum population; *p* is the shape parameter; *δ* is the scale parameter that represents the time needed for the first decimal reduction. The time when *N_t_* reaches the detection limit (100 CFU g^−1^) of the culturable *E. coli* O157:H7, *t_d_*, can also be calculated from Equation (1).

In addition, principal component analysis (PCA) of the parameters (*p* and *δ*) and *t_d_* values were performed using the R software vegan package v 2.0–5 [34] to visualize survival patterns of *E. coli* O157:H7 in the test soils. Stepwise multiple-linear regression analysis was carried out by using SPSS 13.0 for Windows (SPSS Inc., IL, USA) to better understand how soil properties affected the survival of *E. coli* O157: H7 in soils. Analysis of variance (ANOVA) was also carried out to test the differences at the 5% significant level in the survival parameters (*p* and *δ*) and *t_d_* values among soils by SPSS statistic software.

## Results

### Survival of *E. coli* O157:H7 in soils

**Figure 1:**
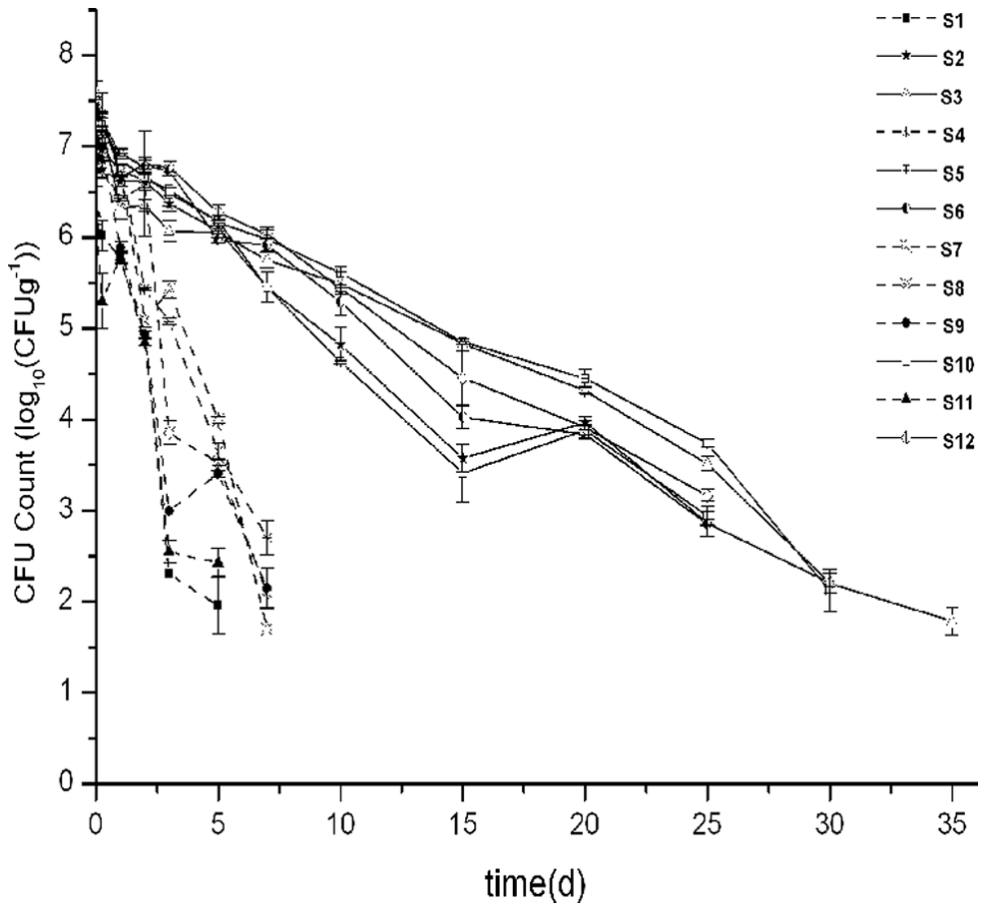
Survival of *E. coli* O157:H7 in the tested soils. Solid line: neutral soils; dashed line: acidic soils.

The *E. coli* O157:H7 population declined by 1.14 log_10_ (CFU g^−1^) within the first day after inoculation in all the test soils. After 1 day, two different survival dynamics of *E. coli* O157:H7 were observed (Fig. 1). *E. coli* O157:H7 declined rapidly in the acidic soils (S1, 4, 7, 8, 9, 11) and was not detectable after 7 days. In the neutral soils (S2, 3, 5, 6, 10, 12), *E. coli* O157:H7 declined much more slowly during 1 – 3 days, and then entered a relatively rapid decline period, reaching the detection limit after 30 days.

### Modeling of survival data

The statistical measures and parameter values of the fitted Weibull model describing the survival of *E*. *coli* O157:H7 in the test soils are presented in Table 2. The *E. coli* O157:H7 survival dynamics in all soils were accurately described by the model, with *R^2^* ranging from 0.84 to 0.99 (P < 0.001), while the first decimal reduction time (*δ*) and the shape parameter (*p*) varied in the test soils (Table 2). Survival times (*t_d_*) of *E*. *coli* O157:H7 in the 12 soils were calculated from the Weibull model (Equation 1). The survival times (*t_d_* ) varied considerably among the soils, with *t_d_* of 6.50±1.34 days in acidic soils with pH of 4.57 to 5.14 and significantly longer (32.79±1.16 days, P < 0.05) in neutral soils (pH, 6.57–7.39). A long survival time of 32.07 days was detected in soil from Xuzhou (S2), where the biggest infection ever reported in China.

**Table 2:** Statistical measures and fitted parameter values of the Weibull model describing the survival of *E*. *coli* O157:H7 in soils.

Soil Code.	*t_d_* #	*δ*	*p*	*R^2^*
S1	4.57±0.23e*	1.01±0.04ef	0.97±0.06ab	0.89±0.01
S2	32.07±0.43c	3.25±0.58cd	0.72±0.05def	0.97±0.01
S3	34.34±0.86a	5.76±0.66b	0.90±0.06ebc	0.97±0.01
S4	8.09±0.35d	0.97±0.09ef	0.80±0.04cd	0.99±0.01
S5	34.03±1.60ab	6.78±1.00a	1.01±0.11ab	0.97±0.01
S6	31.55±0.89c	3.50±0.38c	0.77±0.04cde	0.99±0.01
S7	7.01±0.23d	1.36±0.37ef	1.00±0.17ab	0.91±0.01
S8	7.15±0.22d	1.57±0.14e	1.08±0.06a	0.96±0.01
S9	7.00±0.22d	0.55±0.05f	0.64±0.01f	0.91±0.01
S10	31.96±1.02c	2.56±0.08d	0.67±0.02ef	0.96±0.02
S11	5.16±0.12e	1.03±0.07ef	0.88±0.04bc	0.84±0.01
S12	32.77±1.16bc	5.22±0.43b	0.90±0.04bc	0.98±0.01

# Survival time to reach detection limit (*t_d_*); time needed for first decimal reduction in *E*. *coli* O157:H7 population (*δ*); shape parameter (*p*).

* Significant differences (p < 0.05) indicated by different letters.

### The relationship between soil properties and the survival of *E. coli* O157:H7

For all soils, stepwise regression analysis showed that soil pH, the ratio of G^−^ bacteria PLFAs to G^+^ bacteria PLFAs (G^−^/G^+^); exchangeable K and OC were the key factors affecting the survival of *E. coli* O157:H7. Soil pH (*P* < 0.001) and OC (*P* < 0.05) had positive effects on *E. coli* O157:H7 survival time (*t_d_*). In contrast, a high G^−^/G^+^ ratio, and high exchangeable potassium concentration decreased the survival time. The results also suggested that soil pH and G^−^/G^+^ were the most important factors determining the survival of *E. coli* O157:H7 in the test soils (Table 3).

**Table 3:** Stepwise multiple-linear regression analysis of soil properties and the survival time (*t_d_*) of *E. coli* O157:H7 in the test soils.

			T value of the partial regression coefficient	
Regression equations #	R^2^	F value		T value
			Constant	–13.208
*t_d_* = –34.188+12.360pH –17.856(G^−^/G^+^)−0.076K+0.301OC	0.998	451.782^***^	pH	32.277^***^
			G−/G+	−10.970^**^
			K	−6.450^***^
			OC	6.494^***^

# Survival time to reach detection limit (*t_d_*), the ratio of Gram-negative bacteria phospholipid fatty acids (PLFAs) to Gram-positive bacteria PLFAs (G^−^/G^+^); exchangeable potassium (K); total soil organic carbon (OC); correlation is significant at the 0.001 probability level(***).

## Discussion

**Figure 2:**
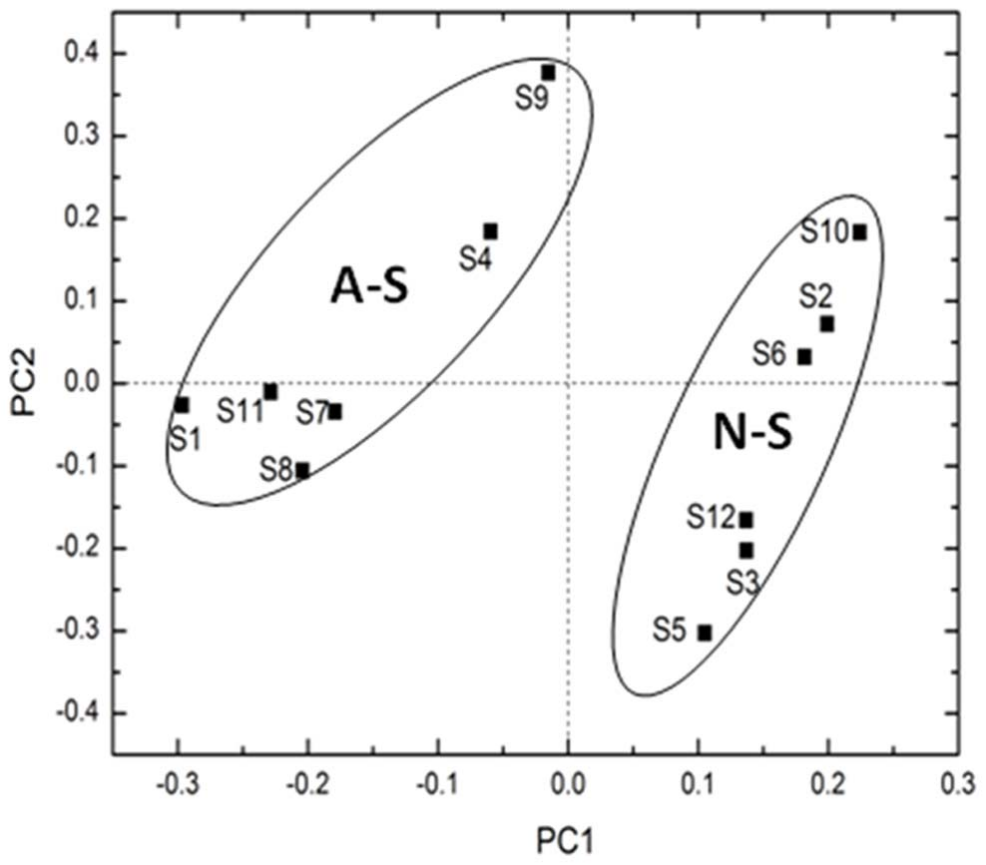
Principal component analysis (PCA) of the survival parameters (*δ*, *p* and *t_d_*). *δ*, *p* and *t_d_* are the same as shown in Table 2. A-S: acidic soils; N-S: neutral and slight alkaline soils.

The results indicated that *E. coli* O157:H7 survival times (*t_d_*) mainly depended on the initial soil pH. Principal component analysis (PCA) clearly showed a substantial difference in *E. coli* O157:H7 survival times between acidic and neutral soils (Fig. 2). In the acidic soils, *E. coli* O157:H7 could not be detected by 7 days after inoculation (Fig.1). However, in the neutral soils, *E. coli* O157:H7 declined much slowly than in the acidic soils and survived for up to 38 days. There have been previous reports which linked *E. coli* O157:H7 survival times (*t_d_*) with soil pH [9], [12], [32], [35], However, our data showed an exceptionally highly statistically significant correlation between these two parameters. It therefore appears that the different physical, chemical and biological properties of acidic and neutral soils resulted in the remarkable differences in *E. coli* O157:H7 survival. Stepwise multiple regression analysis indicated that *E. coli* O157:H7 survival time was significantly and positively correlated with soil pH and OC (Table 3). In addition, the initial G^−^/G^+^ and exchangeable K were negatively correlated with *E. coli* O157:H7 survival time (*t_d_*).

There may be several reasons why soil pH had the most significant positive correlation with *E. coli* O157:H7 survival time (*t_d_*). Firstly, most soil microbial species are generally adapted to neutral or slightly alkaline environments and the quantity of soil bacteria increases from pH 4 to 7, and they can readily adapt to changing pH within this range [36], [37]. Secondly, pH can affect the adsorption of bacteria to soil minerals. The adsorption decreased gradually with increasing pH [38], [39]. For example, Zhao et al. (2013) found that more *E*. *coli* was absorbed by soil colloids when solution pH decreased from 9 to 4 [38]. Also, Cai et al. (2013) showed that large decreases in the viability of *E*. *coli* O157:H7 can be caused by the sorption of *E*. *coli* O157:H7 to soil minerals [40]. Therefore, a large amount of *E. coli* O157:H7 adsorbed to soil minerals would result in a high loss of viability of *E. coli* O157:H7 in low pH soils. Also, the low biological availability of phosphorus and organic nitrogen and the high toxicity of Al and Mn in the soil with low pH [1], [41]–[43], might indirectly affect *E. coli* O157:H7 survival and activity in the acidic soils.

Because of competition for nutrients and niche space, as well as predation, the survival of introduced pathogens can be affected by the diverse coexisting populations of the indigenous microorganisms [9], [15]. The stepwise multiple regression analysis showed that the initial G^−^/G^+^ ratio was negatively correlated with *E. coli* O157:H7 survival time (*t_d_*) (Table 3). van Elsas et al. (2012) and Ma et al. (2013) also indicated that survival of the invading *E. coli* O157:H7 was negatively correlated with soil microbial diversity, due to the competition between native bacterial communities and the introduced species for nutrients and niche space [15], [44]. The G^−^ bacteria are known to out-compete G^+^ bacteria for nutrients in soil [45]. Furthermore, the production of bacteriocins to kill closely related species drives the negative interspecies inter-actions in bacterial systems, which plays an important role in determining the fate of invading bacteria [46]. *E. coli* O157:H7 belongs to G^−^ bacteria, therefore we surmised that G^−^ bacterial species in soil would have a stronger antagonism to *E. coli* O157:H7 than G^+^ bacteria. Another study confirmed that *Bacteroidetes*, *Gammaproteobacteria* and *Firmicutes*, mainly G^−^ bacteria, could inhibit *E*. *coli* O157:H7 survival due to antagonism [47]. Therefore, the greater direct or indirect antagonism of G^−^ bacteria than G^+^ bacteria to *E. coli* O157:H7 might account for the statistically significant negative correlation between *E. coli* O157:H7 survival time (*t_d_*) and the initial G^−^/G^+^ ratio (Table 3).

*E*. *coli* O157:H7 survival times in the test soils were decreased by increasing exchangeable K. A recent study revealed that *t_d_* values decreased significantly (*P*< 0.05) with increasing electrical conductivity and concentrations of individual soil cations, e.g. K^+^, Na^+^, Ca^2+^, and Mg^2+^. This could interfere with ion transport, enzyme activity, and crucial protein synthesis in *E. coli* O157:H7, and finally result in reduced *E. coli* O157:H7 survival in soils [33]. In addition, our results showed that soil OC was significantly correlated with *E. coli* O157:H7 survival time (*t_d_*). Soil organic matter is a major energy source for microorganisms, and can provide carbon sources for the growth and survival of *E. coli* in soil and water [48]. Furthermore, abundant organic carbon can decrease the competitive pressure between organisms through providing easily available energy sources in soil, and thus possibly enhance the persistence of *E. coli* O157:H7 [15]. Soil organic carbon also helps to improve soil structure by forming multi-pored aggregates which serves as microbial habitats [49]. Therefore, the abundance of organic carbon in soil can provide more nutrients, water, air and biological niches for *E. coli* O157:H7 and decrease the competition with indigenous microorganisms, slowing the decline of *E. coli* O157:H7 [3], [15].

## Conclusions

This research has enhanced our understanding of the survival of *E. coli* O157:H7 in soils. *E. coli* O157:H7 could survive for 32.79±1.16 days in neutral soils, and only 6.5±1.34 days in acidic soils. Special attention should be paid to the different survival times of *E*. *coli* O157:H7 in acidic and neutral soils when evaluating the environmental risk associated with it. The survival of *E. coli* O157:H7 in soils might relate to the interactions between numerous physical, chemical and biological factors. Our findings suggested that high soil pH and organic carbon could prolong *E. coli* O157:H7 survival times (*t_d_*), while the initial G^−^/G^+^ ratio and exchangeable K were negatively correlated with survival times. Soil pH governs the fate of *E. coli* O157:H7 directly and indirectly through the adsorption/desorption of soil minerals, nutrition availability, and metal toxicity. However, the experiment was carried out under laboratory conditions. In order to better understand the survival dynamics of *E. coli* O157:H7 and provide precise information to assess the possible risk of contamination by this pathogen, further research should be done under natural conditions.

## References

[1] Ogden ID, Fenlon DR, Vinten AJA, Lewis D (2001) The fate of *Escherichia coli* O157 in soil and its potential to contaminate drinking water. Int J Food Microbiol 66: 111–117. 11407540 10.1016/s0168-1605(00)00508-0

[2] Franz E, Semenov AV, Termorshuizen AJ, de Vos OJ, Bokhorst JG, et al (2008) Manure-amended soil characteristics affecting the survival of *E. coli* O157:H7 in 36 Dutch soils. Environ Microbiol 10: 313–327. 18199123 10.1111/j.1462-2920.2007.01453.x

[3] Williams AP, Avery LM, Killham K, Jones DL (2007) Survival of *Escherichia coli* O157:H7 in the rhizosphere of maize grown in waste amended soil. J Appl Microbiol 102: 319–326. 17241336 10.1111/j.1365-2672.2006.03104.x

[4] Mukherjee A, Cho S, Scheftel J, Jawahir S, Smith K, et al (2006) Soil survival of *Escherichia coli* O157:H7 acquired by a child from garden soil recently fertilized with cattle manure. J Appl Microbiol 101: 429–436. 16882151 10.1111/j.1365-2672.2006.02913.x

[5] Ongeng D, Muyanja C, Ryckeboer J, Geeraerd AH, Springael D (2011) Rhizosphere effect on survival of *Escherichia coli* O157:H7 and *Salmonella enteric serovar typhimurium* in manure-amended soil during cabbage (Brassica oleracea) cultivation under tropical field conditions in sub-Saharan Africa. Int J Food Microbiol 149: 133–142. 21741722 10.1016/j.ijfoodmicro.2011.06.009

[6] Doyle MP, Erickson MC (2008) Summer meeting 2007–the problems with fresh produce: an overview. J Appl Microbiol 105: 317–330. 18284485 10.1111/j.1365-2672.2008.03746.x

[7] Scallan E, Hoekstra RM, Angulo FJ, Tauxe RV, Widdowson MA, et al (2011) Foodborne illness acquired in the United States—major pathogens. Emerg Infect Dis. 17: 7. 10.3201/eid1701.P11101 PMC3375761 21192848

[8] Wang G, Doyle MP (1998) Survival of enterohemorrhagic Escherichia coli O157: H7 in water. J Food Protect 6: 662–667. 10.4315/0362-028x-61.6.662 9709245

[9] Yao ZY, Wei G, Wang HZ, Wu LS, W JJ, et al (2013) Survival of *Escherichia coli* O157:H7 in soils from vegetable fields with different cultivation patterns. Appl Environ Microb 79: 1755–1756. 10.1128/AEM.03605-12 PMC3591966 23291546

[10] Vidovic S, Block HC, Korber DR (2007) Effect of soil composition, temperature, indigenous microflora, and environmental conditions on the survival of *Escherichia coli* O157:H7. Can J Microbiol 53: 822–829. 17898837 10.1139/W07-041

[11] Mubiru DN, Coyne MS, Grove JH (2000) Mortality of *Escherichia coli* O157:H7 in two soils with different physical and chemical properties. J Environ Qual 29: 1821–1825.

[12] Ongeng D, Muyanja C, Geeraerd AH, Springael D, Ryckeboer J (2011) Survival of *Escherichia coli* O157:H7 and *Salmonella enteric serovar Typhimurium* in manure and manure-amended soil under tropical climatic conditions in Sub-Saharan Africa. J Appl Microbiol 110: 1007–1022. 21276146 10.1111/j.1365-2672.2011.04956.x

[13] Jiang XP, Morgan J, Doyle MP (2002) Fate of *Escherichia coli* O157:H7 in manure- amended soil. Appl Environ Microb 68: 2605–2609. 10.1128/AEM.68.5.2605-2609.2002 PMC127522 11976144

[14] Semenov AV, van Overbeek L, Termorshuizen AJ, van Bruggen AHC (2011) Influence of aerobic and anaerobic conditions on survival of *Escherichia coli* O157:H7 and *Salmonella enteric serovar Typhimurium* in Luriae-Bertani broth, farm-yard manure and slurry. J Environ Manage 92: 780–787. 21035246 10.1016/j.jenvman.2010.10.031

[15] van Elsas JD, Chiurazzia M, Mallona CA, Elhottova D, Krištufekb V, et al (2012) Microbial diversity determines the invasion of soil by a bacterial pathogen. Proc Natl Acad Sci USA 109: 1159–1164. 22232669 10.1073/pnas.1109326109 PMC3268289

[16] Li HW, Jing HQ, Pang B, Zhao GF, Yang JC, et al (2002) Study on diarrhea disease caused by enterohemorrhagic *Escherichia coli O157:H7* in Xuzhou city, Jiangsu province in 2000. Chin J Epidemiol 23: 119–122 (in Chinese). 12015094

[17] Liu JB, Yang JC, Jing HQ, Xu JG (2007) Epidemiological investigation of enterohemorrhagic *Escherichia coli* O157:H7 infection status in Xuzhou city of Jiangsu province from 1999 to 2006. Dis Surveill 22: 516–518 (in Chinese).

[18] Xu JG, Quan TS, Xiao DL, Fan TR, Li LM, et al (1990) Isolation and characterization of *Escherichia coli* O157:H7 strains in China. Curr Microbiol 20: 299–303.

[19] Liu JB, Jing HQ, Xu JG (2007) Research progress in enterohemorrhagic *Escherichia coli O157:H7* infection. Chin J School Docto 21: 720–722 (in Chinese).

[20] Zhu WG, Xie SQ, Hong JX, Cui JF, Guo XF (2006) Detection of *Escherichia coli* O157:H7 by multiplex polymerase chain reaction. Chin J Zoono 22: 428–432 (in Chinese).

[21] Wu EP, Liu LZ, Zhang Y (2006) Investigation into the infectiou of *Escherichia coli* O157:H7 in Zhengzhou in 2005. Mod Prev Med 13: 2455–2556 (in Chinese).

[22] Miao QL, Pan WZ, Xu XZ (2008) Characteristic analysis of summer temperature in Nanjing during 56 years. J Tro Meteorol 24: 737–742 (in Chinese).

[23] Richards LA (1949) Methods of measuring soil moisture tension. Soil Sci 68: 95–112.

[24] Agricultural Chemistry Committee of China (1983) Conventional Methods of Soil and Agricultural Chemistry Analysis: Science Press, Beijing, China. (in Chinese)

[25] Wu YP, Ma B, Zhou L, Wang HZ, Xu JM, et al (2009) Changes in the soil microbial community structure with latitude in eastern China, based on phospholipid fatty acid analysis. Appl Soil Ecol 43: 234–240.

[26] Zelles L (1999) Fatty acid patterns of phospholipids and lipopolysaccharides in the characterisation of microbial communities in soil: a review. Biol Fertil Soils 29: 111–129.

[27] Swallow M, Quideau SA (2013) Moisture effects on microbial communities in boreal forest floors are stand-dependent. Appl Soil Ecol 63: 120–126.

[28] Dangi SR, Stahl PD, Wick AF, Ingram LJ, Buyer JS (2012) Soil microbial community recovery in reclaimed soils on a surface coal mine site. Soil Sci Soc Am J 76: 915–924.

[29] Ding N, Guo HC, Hayat T, Wu YP, Xu JM (2009) Microbial community structure changes during Aroclor 1242 degradation in the rhizosphere of ryegrass (Lolium multiflorum L.). FEMS Microbiol Ecol 70: 305–314. 10.1111/j.1574-6941.2009.00742.x 19663919

[30] Ying JY, Zhang LM, Wei WX, He JZ (2013) Effects of land utilization patterns on soil microbial communities in an acid red soil based on DNA and PLFA analyses. J Soil Sediment 13: 1223–1231.

[31] Institute of Soil Science, Academia Sinica (1990) Soils of China. Science Press, Beijing, China. 514–532. (in Chinese)

[32] Wang HZ, Zhang TX, Wei G, Wu LS, Wu JJ, Xu JM. (2013) Survival of *Escherichia coli* O157: H7 in soils under different land use types. Environ Sci Pollut Res. DOI 10.1007/s11356-013-1938-9 10.1007/s11356-013-1938-9 23812736

[33] Ma JC, Ibekwe AM, Crowley DE, Yang CH (2012) Persistence of *Escherichia coli* O157:H7 in major leafy green producing soils. Environ Sci Technol 46: 12154–12161. 23030401 10.1021/es302738z

[34] Oksanen J, Blanchet FG, Kindt R, Legendre P, Minchin PR, et al. (2012) vegan: Community ecology package. R package version 2.0-5. http://CRAN.R-project.org/package=vegan.

[35] Benjamin MM, Datta AR (1995) Acid tolerance of enterohemorrhagic *Escherichia coli* . Appl Environ Microb 61: 1669–1672. 10.1128/aem.61.4.1669-1672.1995 PMC167428 7747983

[36] Fierer N, Jackson RB (2006) The diversity and biogeography of soil bacterial communities. Proc Natl Acad Sci USA 103: 626–631. 16407148 10.1073/pnas.0507535103 PMC1334650

[37] Lauber CL, Hamady M, Knight R, Fierer N (2009) Pyrosequencing-based assessment of soil pH as a predictor of soil bacterial community structure at the continental scale. Appl Environ Microb 75: 5111–5120. 10.1128/AEM.00335-09 PMC2725504 19502440

[38] Zhao WQ, Liu X, Cai P, Huang QY (2013) Mechanisms of bacterial pathogens adsorption on red soil colloids. Acta Pedol Sin 50: 1–9 (in Chinese).

[39] Yee N, Fein JB, Daughne CJ (2000) Experimental study of the pH, ionic strength, and reversibility bacteria–mineral adsorption. Geochim Cosmochim Ac 64: : 609– 617.

[40] Cai P, Huang QY, Walker SL (2013) Deposition and survival of *Escherichia coli* O157:H7 on Clay Minerals in a Parallel Plate Flow System. Environ Sci Technol 47: 1896–1903. 23346967 10.1021/es304686a

[41] Devau N, Cadre EL, Hinsinger P, Jaillard B, Gérard F (2009) Soil pH controls the environmental availability of phosphorus: experimental and mechanistic modelling approaches. Appl Geochem 24: 2163–2174.

[42] Curtin D, Campbell CA, Jalil A (1998) Effects of acidity on mineralization: pH-dependence of organic matter mineralization in weakly acidic soils. Soil Biol Biochem 30: 57–64.

[43] Aciego Pietri JC, Brookes PC (2008) Relationships between soil pH and microbial properties in a UK arable soil. Soil Biol Biochem 40: 1856–1861.

[44] Ma JC, Ibekwe AM, Yang C, Crowley DE (2013) Influence of bacterial communities based on 454-pyrosequencing on the survival of *Escherichia coli* O157: H7 in soils. FEMS Microbial Ecol. 84: 542–554. 10.1111/1574-6941.12083 23360569

[45] Birk JJ, Steiner C, Teixiera WC, Zech W, Glaser B. (2009) Microbial response to charcoal amendments and fertilization of a highly weathered tropical soil [M]//Amazonian Dark Earths: Wim Sombroek's Vision. Springer Netherlands,: 309–324.

[46] Majeed H, Gillor O, Kerr B, Riley MA (2011) Competitive interactions in *Escherichia coli* populations: the role of bacteriocins. ISME J 5: 71–81. 20664553 10.1038/ismej.2010.90 PMC3105674

[47] Westphal A, Williams ML, Baysal-Gurel F, LeJeune JT, Gardener BBM (2011) General suppression of *Escherichia coli* O157:H7 in sand-based dairy livestock bedding. Appl Environ Microb 77: 2113–2121. 10.1128/AEM.01655-10 PMC3067323 21257815

[48] van Elsas JD, Semenov AV, Costa R, Trevors JT (2011) Survival of *Escherichia coli* in the environment: fundamental and public health aspects. ISME J 5: 173–183. 20574458 10.1038/ismej.2010.80 PMC3105702

[49] Simonetti G, Francioso O, Nardi S, Berti A, Brugnoli E, et al (2011) Characterization of humic carbon in soil aggregates in a long-term experiment with manure and mineral fertilization. Soil Sci 76: 880–890.

